# Population trends in the 10-year incidence and prevalence of diabetic retinopathy in the UK: a cohort study in the Clinical Practice Research Datalink 2004–2014

**DOI:** 10.1136/bmjopen-2016-014444

**Published:** 2017-02-28

**Authors:** Rohini Mathur, Krishnan Bhaskaran, Emma Edwards, Helen Lee, Nishi Chaturvedi, Liam Smeeth, Ian Douglas

**Affiliations:** 1 Department of Non-communicable Disease Epidemiology, London School of Hygiene and Tropical Medicine, London, UK; 2 Royal National Institute of Blind People, London, UK; 3 Institute of Cardiovascular Science, UCL, London, UK

**Keywords:** EPIDEMIOLOGY, PRIMARY CARE, PUBLIC HEALTH

## Abstract

**Objectives:**

To describe trends in the incidence and prevalence of diabetic retinopathy (DR) in the UK by diabetes type, age, sex, ethnicity, deprivation, region and calendar year.

**Design:**

Cohort study using the Clinical Practice Research Datalink (CPRD).

**Setting:**

UK primary care.

**Participants:**

7.7 million patients ≥12 contributing to the CPRD from 2004 to 2014.

**Primary and secondary outcome measures:**

Age-standardised prevalence and incidence of diabetes, DR and severe DR (requiring photocoagulation) by calendar year and population subgroup. Relative risk of developing DR and severe DR by population subgroup.

**Results:**

The prevalence of DR was 48.4% in the population type 1 diabetes mellitus (T1DM) (14 846/30 657) and 28.3% (95 807/338 390) in the population with type 2 diabetes mellitus (T2DM). Prevalence of DR remained stable in people with T2DM and decreased in people with T1DM. Screening for DR increased over time for patients with T2DM and remained static for patients with T1DM Incidence of DR increased in parallel with the incidence of T2DM in both diabetic populations. Among patients with T2DM, relative risk of DR varied significantly by region, was increased for older age groups and in men compared with women, with risk of severe DR increased in South Asian groups and more deprived groups. Relative risk of DR for patients with T1DM varied by age and region, but not by gender, ethnic group or deprivation.

**Conclusions:**

This is the largest study to date examining the burden of DR in the UK. Regional disparities in incidence may relate to differences in screening delivery and disease ascertainment. Evidence that deprivation and ethnicity are associated with a higher risk of severe DR highlights a significant potential health inequality. Findings from this study will have implications for professionals working in the diabetes and sight loss sectors, particularly to inform approaches for diagnosis of retinopathy and campaigning to better tackle the disease for at risk groups.

**Strengths and limitations of this study**

- This study constitutes the largest ever sample size to examine trends in the burden of diabetes and diabetic retinopathy (DR) in the UK which allowed for sufficient power to detect relationships between population subgroups, which is often unfeasible in smaller studies where population sizes do not allow for such granular comparisons.
- Since recording of screening of DR was incentivised under Quality and Outcomes Framework (QOF) from 2004 to 2014 and QOF indicators are known to be well recorded by general practitioners and so we anticipate that screening and identification of DR will have been recorded with a high degree of accuracy during the study period.
- This study relied on coded diagnoses of diabetes and retinopathy as we did not have access to data from other sources such as retinal photography or practitioner letters, which could have been used to validate the diagnoses.

## Introduction

Diabetic retinopathy (DR) is the most common form of eye disease among individuals with diabetes mellitus. In the UK, within 20 years of diabetes diagnosis nearly all people with type 1 and almost two-thirds of people with type 2 diabetes (60%) have some degree of retinopathy.^1^ ^2^ DR is one of the leading causes of visual impairment and blindness in the UK, among people of working age; compared with the general population, risks of cataract and of glaucoma are doubled among individuals with established DR.^3^

DR is a progressive disease directly attributable to diabetes, which affects the blood vessels of the retina. The blood vessels can leak, become blocked or proliferate excessively.^4^ ^5^ If untreated, this can lead to retinal damage and visual impairment.^6^

Differences in the risk of diabetes by gender, ethnic group and deprivation have been established in the UK and worldwide.^7–11^ In the UK, the risk of developing type 2 diabetes is elevated approximately twofold in South Asian and African Caribbean communities compared with the white British population.^12–14^ Ethnic differences in diabetes are mirrored by ethnic differences in DR in the UK and globally.^15^ ^16^ In the USA, several studies have reported differences between white, black, Hispanic and Mexican American populations.^17–20^ For example, the National Health and Nutrition Examination Survey reported the prevalence of DR to be 36% higher in black and 84% higher in Mexican American populations relative to the white population with diabetes.^21^ UK studies have demonstrated higher prevalence of DR among individuals of South Asian ethnicity relative to white.^16^ ^22^ Socioeconomic deprivation has also been found to be associated with DR, with the UK and international studies reporting higher prevalence among more deprived groups.^23^ ^24^

In order to prevent, delay and better manage DR, annual screening using digital photography is recommended for all people with diabetes aged 12 and over in the UK. Introduced in 2004, uptake of the screening programme has increased steadily, achieving full national coverage in 2008.^25^ Implementation of screening varies across each of the four devolved nations of the UK; Typically, all people with diabetes aged 12 and over are invited for a screening appointment via letter or phone call, which can take place in general practices, hospitals, at specialist diabetes clinics, mobile clinics or at the high street opticians. Retinal photography to assess grade of retinopathy is completed. If the DR is considered to be sight threatening, the individual is referred to hospital eye services for treatment, otherwise the results are sent back to the general practitioner for continuing diabetes care.^26^ ^27^

Despite extensive literature detailing the prevalence and incidence of diabetes in the UK, population-wide measures of incidence and prevalence of DR in the UK context have not been determined. Previous UK focussed research on retinopathy has largely been limited to estimates based on regional screening programmes or small general practices samples.^28–32^ Having a more complete understanding of the burden of disease due to DR across the diverse UK population will help improve future service planning and provision of preventive and therapeutic care. The aim of this study was to generate nationally representative estimates of the incidence and prevalence of DR in the UK between 2004 and 2014 using the Clinical Practice Research Datalink (CPRD), and to examine trends in the prevalence and relative risk of retinopathy by diabetes type, age, sex, ethnicity, socioeconomic deprivation and region.

## Methods

### Data sources

The CPRD is an electronic health database which currently contains longitudinal primary care records for ∼13.5 million patients from 601 general practices across the UK (covering 7% of the UK population), of whom 5.5 million are currently active.^33^ The CPRD contains anonymised patient-level information on demographics, lifestyle data, clinical diagnoses, prescriptions and preventive care. The database was established in 1987, and continuous observational data have been collected in most practices for over 6 years yielding over 30 million patient years of observation. Data undergo regular quality checks and practices are deemed to be ‘up to standard’ if their data are deemed to be of research-level quality.^33^ The CPRD has been found to be representative of the UK population with respect to gender, age and ethnic group.^33^ ^34^

### Identification of DR

Within the CPRD, diagnoses and symptoms are coded using the Read clinical hierarchy, which is the coding standard used across the UK primary care.^35^ Clinical terms to identify diagnoses of DR were agreed on via consultation between the research team and clinicians. All diagnoses of DR were identified by searching for Read clinical terms in the CPRD. DR was classified as severe if the codes pertained to laser therapy, advanced retinopathy or proliferative retinopathy.

Onset of DR was defined as the first ever diagnostic Read code entered onto the patient record. Patients with a diagnosis for severe retinopathy at any time were included in a subanalysis of patients with advanced disease, with onset defined as the earliest ever code of severe DR on the patient record.

Screening for DR was identified using a set of clinical terms which indicated that a screening event had occurred. Codes indicating that an individual had been invited to or referred for screening were not included. A summary of the clinical terms used to identify DR and screening can be found in the online supplementary material table S1.

10.1136/bmjopen-2016-014444.supp1 supplementary data

### Identification of diabetes mellitus

For the purposes of this study, classification of patients into categories of type 1 diabetes or type 2 diabetes was determined using algorithms developed by the UK Biobank Study for use in electronic health records.^36^ The algorithms initially classify patients according to the presence of diagnostic Read codes for type 1 or type 2 diabetes. The diagnoses are then confirmed if supporting information such as prescriptions of antidiabetic medications, raised blood glucose and diabetes process of care measures are present. All individuals identified as having type 1 or type 2 diabetes after successfully passing through the adjudication algorithm were included in the final analysis.

### Covariate definition

Age was grouped into 10-year age bands. Deprivation was classified using the Index of Multiple Deprivation (IMD) and divided into quintiles.^37^ Each patient in the study was assigned a deprivation score relating to the deprivation value of their general practice. Information on ethnic group was derived from the CPRD record where available and updated using ethnicity recorded in linked Hospital Episode Statistics data if missing in CPRD. Conflicts between the two data sources were resolved using a defined and previously validated algorithm.^38^ Ethnicity was grouped into the five categories of the 2011 census, namely, white, South Asian, black African/Caribbean, mixed and other. Patients with missing ethnicity or with codes which were unusable were collapsed into a category of unknown ethnicity (see supplementary material for algorithm and Read codes tables 4–5). Duration of diabetes at onset of DR (expressed in years) was calculated by subtracting the date of the first diagnostic code for diabetes from the date of the first diagnostic code for DR. Age at diabetes onset (expressed in years) was calculated by subtracting the date of birth from the date of diabetes onset.

### Statistical methods

A population-based cohort study was conducted to examine the prevalence and incidence of DR in all patients aged 12 years and over registered with the CPRD between January 2004 and December 2014. The prevalence and incidence of diabetes and DR and severe DR was examined separately for individuals with type 1 diabetes and type 2 diabetes. The age-standardised prevalence of screening in each year was also examined for individuals with type 1 and type 2 diabetes.

All prevalence and incidence estimates were standardised against the mid-2014 UK population estimates from the Office for National Statistics.^39^ The overall age-standardised prevalence of DR stratified by diabetes status, gender, ethnic group, deprivation quintile and region was calculated for the entire study population. For the study of prevalence over time, the outcome was defined as all individuals with a relevant diagnostic code at the midpoint of each calendar year from January 2004 to December 2014. Point prevalence was calculated by dividing the number of individuals with DR, severe DR by the number of patients in the CPRD aged 12 years and over on 1 July of each year. The proportion of patients receiving DR screening in each year was determined by dividing the number of patients with a code for screening in each calendar year by the number of patients in the CPRD as a whole, and with type 1 or type 2 diabetes, aged 12 and over on 1 July of each year.

For the study of disease incidence, the outcome was first diagnosis of DR or severe DR between January 2004 and December 2014. Individuals with a first diagnosis of retinopathy prior to 2004 were excluded from the analysis. Incidence was calculated by dividing the number of newly diagnosed patients aged 12 and over by the number of person-years of follow-up of all eligible patients aged 12 and over contributing to the CPRD for each calendar year. Age standardised incidence rates of DR and severe DR per 10 000 person-years of follow-up time were calculated for all patients in the CPRD for 2014, the final year of the study.

Cox proportional hazards regression was used to evaluate the risk of DR in all patients between January 2004 and December 2014. HRs for the relative risk of DR and severe DR, mutually adjusted for age, gender, deprivation, ethnic group, region and duration of diabetes, were calculated separately for individuals with type 1 and type 2 diabetes. The start of follow-up was defined as the latest of practice ‘up to standard date’ (up to standard indicating the practice data meets the range of quality criteria as defined and applied by CPRD) or 12 months after the patients' current registration date. Follow-up time ended at the earliest date of; first diagnosis of DR, transferring out of the practice, latest data collection, death or 31 December 2014. Stata statistical software V.13 was used for all analyses (StataCorp, Stata Statistical Software: Release 13. 2013).

## Results

From the entire CPRD population of 13.7 million patients, 7 707 475 patients registered with the CPRD between 2004 and 2014, aged 12 and over were eligible for inclusion in the study (see online supplementary supplementary appendix figures 2–5 for full details).

### Overall prevalence of DR

Over the 10-year study period, 79.3% of individuals with type 1 diabetes and 82.6% of individuals with type 2 diabetes had evidence of ever having had a DR screen completed, with over 50% having had their latest screen in the 15 months prior to the end of their follow-up period.

A total of 144 362 prevalent cases of DR were identified between 2004 and 2014, giving a crude 10-year period prevalence of 1.9% in the entire CPRD population, 48.4% in the population with type 1 diabetes and 28.3% in the population with type 2 diabetes. A total of 9085 prevalent cases of severe DR were identified during the study period. The crude 10-year period prevalence of severe DR was 0.1% in the CPRD population, 7.0% in the population with type 1 diabetes and 1.4% in the population with type 2 diabetes (table 1).

**Table 1:** Demographic characteristics of the CPRD population registered between 2004 and 2014

	All CPRD patients	T1DM patients	T2DM patients			
Population*	N	%	N	Per cent	N	Per cent
Total (12+)	7 707 475	100.0%	30 657	100.0%	338 390	100.0%
Gender						
Men	3 790 664	49.2%	17 761	57.9%	187 141	55.3%
Women	3 916 811	50.8%	12 896	42.1%	151 249	44.7%
Ethnic group						
White	4 006 927	52.0%	19 810	64.6%	205 168	60.6%
South Asian	223 090	2.9%	453	1.5%	15 840	4.7%
Black	142 070	1.8%	373	1.2%	7186	2.1%
Other	109 402	1.4%	254	0.8%	3891	1.2%
Mixed	50 363	0.7%	152	0.5%	1095	0.3%
Unknown	3 175 623	41.2%	9615	31.4%	105 210	31.1%
IMD quintile						
1 (most affluent)	1 338 388	17.4%	5280	17.2%	52 280	15.5%
2	1 496 051	19.4%	5934	19.4%	61 008	18.0%
3	1 621 330	21.0%	6517	21.3%	71 982	21.3%
4	1 723 122	22.4%	6910	22.5%	79 130	23.4%
5 (least affluent)	1 470 726	19.1%	5833	19.0%	72 094	21.3%
Region						
North east	121 334	1.6%	516	1.7%	5465	1.6%
North west	803 853	10.4%	3226	10.5%	40 255	11.9%
Yorkshire and the Humber	269 265	3.5%	1050	3.4%	10 888	3.2%
East midlands	296 884	3.9%	1182	3.9%	12 489	3.7%
West midlands	654 656	8.5%	2451	8.0%	30 710	9.1%
East of England	752 786	9.8%	3041	9.9%	29 139	8.6%
South west	651 327	8.5%	2601	8.5%	30 330	9.0%
South central	853 405	11.1%	3242	10.6%	33 416	9.9%
London	1 017 747	13.2%	3150	10.3%	39 281	11.6%
South east	792 775	10.3%	3123	10.2%	33 399	9.9%
Northern Ireland	199 509	2.6%	937	3.1%	9322	2.8%
Scotland	711 397	9.2%	3601	11.7%	31 387	9.3%
Wales	582 537	7.6%	2537	8.3%	32 309	9.5%
Retinopathy screen 2004–2014	710 445	8.7%	24 828	79.3%	279 495	82.6%
Retinopathy screen in last 15 months	336 960	4.1%	15 788	50.4%	180 268	53.3%
Diabetic retinopathy	144 362	1.9%	14 846	48.4%	95 807	28.3%
Severe diabetic retinopathy	9085	0.1%	2148	7.0%	4651	1.4%
Age at diabetes diagnosis (in years, mean, SD)		26	(18)	60	(14)	
Mean duration of diabetes at DR onset (years, SD)		14.7	(12.2)	5.9	(6.9)	
Mean duration of diabetes at severe DR onset (years, SD)	20.9	(12.7)	10.4	(8.7)		

*All columns refer to number and %, unless otherwise specified.

CPRD, Clinical Practice Research Datalink; DR, diabetic retinopathy; IMD, Index of Multiple Deprivation; T1DM, type 1 diabetes mellitus; T2DM, type 2 diabetes mellitus.

Mean duration of diabetes at time of DR onset ranged from 6 years for people with type 2 diabetes to 15 years for people with type 1 diabetes, with duration ∼5 years longer for onset of severe DR.

### Age-standardised prevalence and incidence of DR and screening over time

In the whole CPRD population, the age-standardised prevalence of DR decreased over time from 2.6% to 2.2% while the age-standardised prevalence of severe DR remained stable at 0.1%. The incidence of DR increased from 12.1 events per 10 000 years in 2004 to a peak of 23.82 events per 10 000 person-years in 2011 before declining again (annual increase of 0.7 events per 10 000 person-years, p=0.062). The incidence of severe DR remained stable at one event per 10 000 person-years (no trend over time, p=0.265).

The age-standardised proportion of patients having received a DR screen in each calendar increased over the 10-year study period for patients with type 2 diabetes from 40.3% in 2004 to 63.9% in 2014 (annual increase of 2.3%, p<0.001) and oscillated between 59 and 67% for patients with type 1 diabetes over the study period (no trend over time, p=0.291).

**Figure 1:**
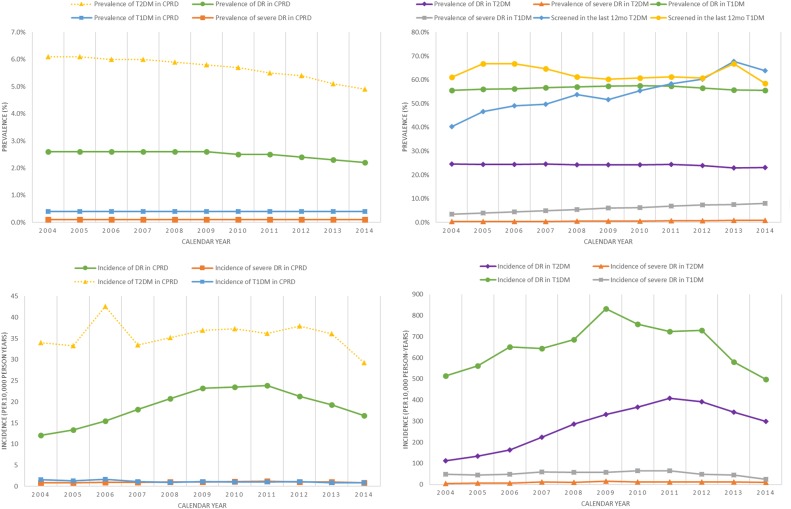
Age-standardised prevalence and incidence of diabetes, screening and diabetic retinopathy 2004–2014. CPRD, Clinical Practice Research Datalink; DR, diabetic retinopathy.

Among patients with type 2 diabetes, the prevalence of DR reduced from 24.6% in 2004 to 23.1% in 2014. The age-standardised incidence of DR increased in parallel with the incidence of type 2 diabetes, increasing from 113.2 events per 10 000 person-years in 2004 to 408.6 events per 10 000 person-years in 2011 and declining thereafter (annual increase of 26 events per 10 000 person-years, 95% CI13.2 to39.3, p=0.001). The age-standardised prevalence of severe DRincreased from 0.3% in 2004 to 0.9% in 2014 (annual increase of 0.06% per year, p<0.001). The age-standardised incidence of severe DRincreased from 5.2 events per 10 000 person-years in 2004 to 10.2 events per 10 000 person-years in 2014 (annual increase of 0.6 events per 10 000 person-years, 95% CI0.01 to1.16, p=0.046) (figure 1).

Among patients with type 1 diabetes the age-standardised prevalence of DR remained stable at 55% (no trend over time p=0.917). The age-standardised incidence of DR increased from 514.7 events per 10 000 person-years in 2004 to 832 events per 10 000 person-years in 2009 and declined thereafter (annual increase of 55.6 events per 10 000 person-years from 2004 to 2009, 95% CI 29.6 to 81.7, p=0.004) The prevalence of severe DR increased from 3.5% in 2004 to 8.0% in 2014 (annual increase of 0.05%, 95% CI 0.04% to 0.05%, p<0.001). The incidence of severe DR decreased non-significantly from 48 events per 10 000 person-years in 2004 to 25.4 events per 10 000 years in 2014 (no trend over time, p=0.459).

### Age-standardised prevalence of DR in 2014

In 2014, the final year of the study period, 73 658 prevalent cases of DR were identified, giving an age-standardised point prevalence of 2.2% ( 95% CI 2.18% to 2.21%) in the entire CPRD population, 54.8% ( 95% CI 53.6% to 56.1%) in the population with type 1 diabetes and 22.7% ( 95% CI 22.0% to 23.8%) in the population with type 2 diabetes (table 2).

**Table 2:** Age-standardised prevalence of diabetic retinopathy in the CPRD 2014

	Diabetic retinopathy						Severe diabetic retinopathy					
	T2DM population			T1DM population			T2DM population			T1DM population		
	160 418			13 848			160 418			13 848		
2014 denominator (12+)	N	%	p Value	N	%	p Value	N	%	p Value	N	%	p Value
Overall												
* Prevalence (95% CI)*	***49 166***	***22.7 (22.0–23.8)***	***7583***	***54.8 (53.6–56.1)***	***1933***	***0.9 (0.8–1.0)***	***1117***	***8.1 (7.6–8.6)***	*** ***	*** ***	*** ***	*** ***
Gender												
* *Men	28 299	23.5	<0.001	4393	54.5	0.916	1206	0.9	<0.001	638	7.9	0.546
* *Women	20 867	21.9		3190	55.3		727	0.8		479	8.4	
Age group												
* *12–34	246	14.3	<0.001	1522	32.9	<0.001	9	0.5	<0.001	125	2.7	<0.001
* *35–44	1307	20.4		1381	58.5		49	0.8		209	8.9	
* *45–54	5039	24.2		1906	65.4		209	1		321	11.0	
* *55–64	10 107	28.6		1376	70.8		437	1.2		222	11.4	
* *65–74	14 837	32.1		888	70.4		620	1.3		162	12.9	
* *75+	17 630	35.3		5104	68.6		609	1.2		78	10.5	
Ethnic group												
* *White	30 253	23.6	<0.001	5028	55.3	<0.001	1105	0.8	<0.001	778	8.5	0.001
* *South Asian	2545	25.2		94	45.2		141	1.1		*	2.5	
* *Black	1190	25.4		59	45.8		75	0.8		10	10.3	
* *Other	605	29.3		47	46.8		21	0.8		*	4.4	
* *Mixed	156	19.8		22	42.2		16	1.8		*	4.3	
* *Unknown	14 417	20.8		2333	55.1		575	1.0		317	7.5	
IMD quintile												
* *1 (most affluent)	7354	20.3	<0.001	1501	55.1	0.010	276	1.0	0.435	226	8.4	0.114
* *2	9780	23.4		1558	54.8		430	0.8		244	8.6	
* *3	9910	23.1		1541	55.9		340	0.8		218	7.7	
* *4	12 144	25.9		1688	55.9		452	0.9		248	8.2	
* *5 (least affluent)	9456	20.6		1233	51.8		398	0.9		174	7.6	
Region												
* *North east	580	26.9	<0.001	73	57.9	<0.001	16	0.7	<0.001	12	9.2	<0.001
* *North west	4846	19.8		677	49.1		175	0.9		102	7.5	
* *Yorkshire and Humber	398	20.4		76	50.2		11	0.8		14	8.4	
* *East midlands	218	44.4		17	68.1		*	0.6		6	24.4	
* *West midlands	4310	22.1		555	55.0		177	0.8		94	9.4	
* *East of England	2563	19.8		494	53.6		119	0.8		66	7.1	
* *South west	4397	26.3		584	56.2		175	1.1		104	9.9	
* *South central	5685	23.9		945	54.2		219	0.8		149	8.3	
* *London	6427	23.8		709	50.3		290	0.9		96	7.0	
* *South east	4635	17.4		773	49.4		157	0.6		103	6.5	
* *Northern Ireland	813	10.6		240	39.7		77	0.7		37	627	
* *Scotland	7930	31.9		1478	66.7		261	1.1		175	8.3	
* *Wales	6634	23.2		962	58.9		254	1.1		159	9.7	

*All figures standardised against the UK mid-2014 population, p values from χ^2^ test for unordered categorical variables, from test for trend for ordered categorical variables (age group and IMD quintile). Table values under 5 are suppressed.

CPRD, Clinical Practice Research Datalink; IMD, Index of Multiple Deprivation; T1DM, type 1 diabetes mellitus; T2DM, type 2 diabetes mellitus.

Among patients with type 2 diabetes, the age-standardised prevalence of DR was higher among men and those of non-white ethnicity and varied substantially by geographic region. Age-standardised prevalence of DR increased with age and with deprivation until quintile 4. The age-standardised prevalence of severe DR was higher for men and those of South Asian and mixed ethnicity. Prevalence increased with age and varied by geographic region, but not by deprivation.

Among patients with type 1 diabetes, the prevalence of DR and severe DR was comparable between men and women and between deprivation quintiles. Prevalence of DR was highest in the white ethnic group compared with all other ethnic groups, while prevalence of severe DR was highest in the black group. Prevalence of DR and severe DR varied substantially by geographic region.

### Relative risk of retinopathy in patients with type 2 diabetes

Median follow-up time for patients with type 2 diabetes was 9.1 years (IQR 5.4–10.9 years). Within this population, fully adjusted HRs from Cox proportional hazards regression showed that the risk of developing DRand severe DRwas reduced in women compared withmen (HR 0.93, 95% CI0.92 to0.95 for DR and HR 0.80, 95% CI0.72 to0.89 for severe DR).

Relative to those aged 55–64, the risk of developing DR was reduced in the youngest and oldest age groups. In the analysis of severe DR, risk was increased in those aged 35–44 and 45–54 relative to those aged 55–64 and reduced in all older age groups.

Each 5-year increase in the duration of diabetes at baseline was associated with a 17% increase in the risk of DR ( 95% CI 1.16 to 1.18) and a 42% increase in the risk of severe DR ( 95% CI 1.39 to 1.45) after adjustment for all other factors.

Risk of DR was equivalent between ethnic groups in the main analysis, and rose for the South Asian group relative to the white group in the analysis of severe DR (HR 1.25 95% CI 1.00 to 1.56).

**Figure 2:**
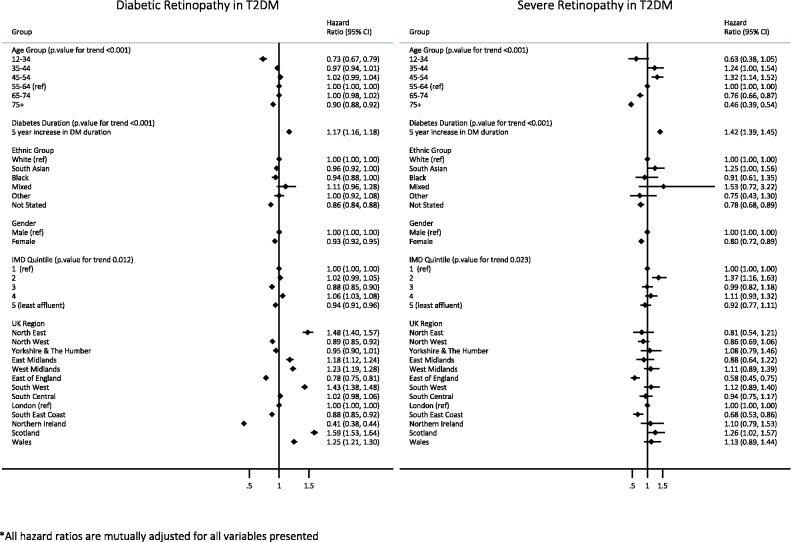
Relative risk of diabetic retinopathy in patients with type 2 diabetes by gender, ethnic group, age group, deprivation, region and duration of diabetes. IMD, Index of Multiple Deprivation; T2DM, type 2 diabetes mellitus.

No clear relationship between deprivation and retinopathy was clear in analysis of all DR. The risk severe DR was raised in the second most affluent group relative to the most affluent group only (HR 1.37 95% CI 1.16 to 1.63) (figure 2).

Risk of DR varied substantially by geographic region, with differences attenuated for severe retinopathy. In comparison to London (the reference region), risk of retinopathy was reduced in Northern Ireland and the east of England, and increased in most other regions of the UK.

### Relative risk of retinopathy in patients with type 1 diabetes

Median follow-up time for patients with type 1 diabetes was 7.1 years (IQR 4.0–10.9 years). Within this population, fully adjusted HRs from Cox proportional hazards regression showed no evidence for differences in the risk of developing DR or severe DR by gender, ethnic group or deprivation.

Relative to those aged 55–64, the risk of developing DR was reduced in the both older age groups. In the analysis of severe DR, risk decreased as age increased.

**Figure 3:**
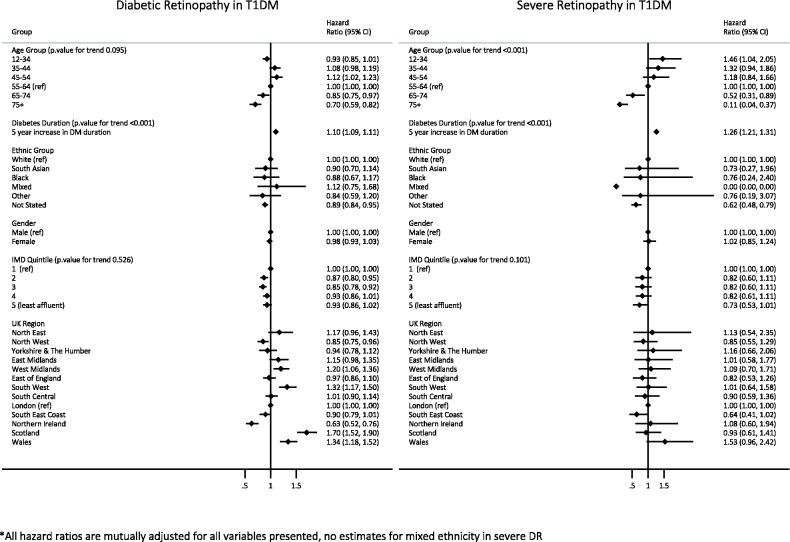
Relative risk of diabetic retinopathy in patients with type 1 diabetes by gender, ethnic group, age group, deprivation, region and duration of diabetes. IMD, Index of Multiple Deprivation; T1DM, type 1 diabetes mellitus.

Each 5-year increase in the duration of diabetes at baseline was associated with a 10% increase in the risk of DR ( 95% CI 1.09 to 1.11) and a 26% increase in the risk of severe DR ( 95% CI 1.21 to 1.31) after adjustment for all other factors. Regional differences in the risk of retinopathy and severe retinopathy for patients with type 1 diabetes mirrored those found in the analysis of patients with type 2 diabetes (figure 3).

## Discussion

### Main findings

The study has shown that while the age-standardised prevalence of DR has remained stable over time for patients with type 1 and type 2 diabetes, the prevalence of severe DR has increased threefold over the 10-year study period. In contrast, while the incidence of DR among people with type 1 and type 2 diabetes has increased to a peak and subsequently decreased, in parallel to the incidence of type 2 diabetes, the incidence of severe DR has increased for patients with type 2 diabetes and halved for patients with type 1 diabetes. The proportion of patients receiving a DR screen in each calendar increased steadily over the 10-year study period for patients with type 2 diabetes to 64% in 2014 and remained stable over time for patients with type 2 diabetes.

The study has further demonstrated that, for individuals with type 2 diabetes, the relative risk of developing DR varies by region, age group and gender, while the relative risk of developing severe retinopathy varies also by ethnicity.

The overall prevalence of DR found in our CPRD population is comparable to that of contemporaneous studies. A 2015 study of the National Diabetic Retinopathy Screening Service for Wales reported the prevalence of DR to be 56% for those with type 1 diabetes and 30.3% for those with type 2 diabetes.^30^ These figures tally closely with the respective 10-year prevalence figures of 48.4% for patients with type 1 diabetes and 28.3% for patients with type 2 diabetes from our study overall, and also for the estimates specific to Wales reported herein (58.9% and 23.2%, respectively). Similarities extend to severe DR also; the same study reports prevalences of 11.2% in those with type 1 diabetes and 2.9% in those with type 2 diabetes. Our study has found the prevalence to be 10.3% and 2.4%, respectively. A recent review of DR studies in western countries has reported the prevalence of DR in to be 28.7% for all people with diabetes, further lending credence to our findings.^40^

The overall incidence of retinopathy increased to a peak partway through the study before decreasing again. Increases in the incidence of retinopathy are likely to be related to increasing incidence of type 2 diabetes and increased ascertainment of retinopathy through nationwide screening programmes, which increased in coverage over the duration of the study period. Annual incidence figures obtained in our study are largely in line with incidence figures reported in the Liverpool Diabetic Eye Study, which examined incidence among patients with type 2 diabetes.^31^

A key finding of the study was large regional variations in the relative incidence of retinopathy, after accounting for age, gender, deprivation and ethnicity. Regional differences in incidence may relate to regional differences in the organisation and delivery of screening programmes, and subsequent ascertainment of disease. It has been suggested that uptake of screening, and as a result, opportunities for diagnosis, may be lower in rural versus urban areas, due to decreased accessibility of screening services.^32^ Qualitative research elucidating the influence of practice-level factors on attendance at screening has also identified challenges in identifying DR including communication with screening services, communication with patients, integration of screening services with other aspects of clinical care and ethnically diverse patient populations.^26^ ^41^

The increased risk of severe DR for South Asian individuals with type 2 diabetes relative to the white group mirrors ethnic differences in diabetes prevalence, and may be due to the same underlying genetic and biological factors which predispose South Asian groups to insulin resistance, as well as cultural factors such as diet.^42^ ^43^ Acculturation to western lifestyles among migrants is also associated with an increase in risk of developing non-communicable diseases, as migrant populations shift towards more sedentary and urbanised lifestyles.^42^ ^44^ Similarly, increased risk of severe DR in the more deprived quintiles relative to the least deprived quintile is consistent with existing literature around socioeconomic disparities in diabetes.^32^ ^45^

Turning to patients with type 1 diabetes, the stability of the prevalence of retinopathy was to be expected as the prevalence and incidence of type 1 diabetes is not subject to large increases resulting from an increasingly obesogenic environment, as is the case with the current epidemic of type 2 diabetes.

The differences in prevalence by gender and ethnic group found here confirm those of recent smaller UK based studies. In 2012, Sivaprasad *et al* reported reduced odds of prevalent retinopathy in women compared with men (OR 0.93, 95% CI 0.90 to 0.97) and raised odds in South Asian and blackAfrican/Caribbean groups compared withwhite (South Asian OR 1.10, 95%CI 1.02 to1.18, blackOR 1.79, 95% CI1.70 to1.89) amongindividuals with diabetes in the UK.^46^

### Strengths

This study made use of high levels of ethnicity recording and linkage with deprivation data provided by the Office for National Statistics (ONS) to describe patterns by ethnicity and IMD. This study constitutes the largest ever sample size to examine trends in the burden of diabetes and DR in the UK. This allowed for sufficient power to detect relationships between populations stratified by gender, ethnic group, geographic region and deprivation, which is often unfeasible in smaller studies where population sizes do not allow for such granular comparisons. At the time of publication, this is the only national study to examine ethnicity and deprivation in relation to the prevalence and incidence of DR.

Since 2004 it has been a requirement of the UK Quality and Outcomes Framework (QOF) that patients with diabetes should be screened annually for DR, and that screening should be recorded by general practitioners in patient records. QOF indicators are known to have been well recorded by general practitioners and so we anticipate results of screening will have been recorded with a high degree of accuracy during the study period.^47^ ^48^

The advantage of routine electronic health databases is that they are regularly updated and can be used to provide timely information on the demographic makeup of the general population and on areas of growing healthcare need.

The CPRD has been used extensively for observational studies examining a wide range of health conditions and the data held within have been widely validated.^33^ The data in the CPRD are prospectively collected and, as a result, the data are not subject to recall bias (the presence of a disease outcome affects the reporting of exposure status) or observer bias (the knowledge of the patient's disease status influences ascertainment or recording of exposure).

### Limitations

The primary purpose of the clinical data held in the CPRD is for patient care, rather than research. By its nature it only includes information gathered at consultation and is thus routinely collected rather than researcher-led. As a result, the completeness and accuracy of data are subject to temporal changes in coding practices, health priorities and population need. Anything not reported to the general practitioner is necessarily not recorded. The absence of a code does not necessarily mean that an individual is free from that condition, but could also be interpreted as being unknown.

Information of ethnicity was missing for 30% of patients with diabetes, which may have resulted in an underestimate of the ethnic differences in incidence and prevalence estimates of DR. In addition to incomplete data, a further potential problem with routine electronic health records is incorrect coding stemming from errors in the way data is entered. A wide range of studies have found the validity of diagnoses and process of care measures in CPRD to be high.^49–51^ Combined with the fact that the CPRD data are subject to ongoing internal quality checks and that concerns with data quality are fed back to the general practices, researchers can be reassured that errors which do occur in the database are kept to a minimum.

This study relied solely on the coded diagnoses of diabetes, retinopathy, eye disease and visual impairment. We did not have access to data from other sources such as retinal photography or practitioner letters, which could have been used to validate the diagnoses.

The use of multiple testing across a range of population subgroups meant that some of the observed associations may have arisen due to chance.

Clinical trials have established duration of diabetes, hyperglycaemia and hypertension as key risk factors in the development of DR.^52^ ^53^ Further work examining the role of pharmacological treatment and risk factor management will be essential in elucidating patterns of DR further, particularly as the UK population ages and the burden of diabetes grows.

### Policy implications

According to the Office for National Statistics, the size of the UK population at the midpoint of 2014 was 64.6 million people.^54^ Given that the CPRD is representative of the UK population structure, we estimate that the absolute number of people with any form of DR in the UK is ∼1.5 million and that the absolute number of people with severe DR is around 140 000. Increases in prevalence of DR are likely to be related to increasing prevalence of type 2 diabetes mellitus and potentially increased ascertainment through national screening programmes.

Findings from the 2013 to 2014 screening programmes in England report highlighted the success of DR screening programmes in reducing the burden of DR in the UK, to the point where, it is now, no longer the leading cause of blindness among working age people in the UK.^55^ In 2014, attendance at DR screening was removed from the QOF, meaning that this important indicator will no longer be collected to such a high accuracy for all diabetic patients. This will impact on future research into retinopathy, and it is likely to have serious negative implications on service planning for diabetic patients unless the indicator is reinstated. Whether the proportion of patients receiving screening decreases from the figures reported in this study after 2014, and the impact this will have on future ascertainment and management of DR will need to be explored.

Findings from this study will have implications for professionals working in the diabetes and sight loss sectors, particularly to inform approaches for diagnosis of retinopathy and campaigning to better tackle the disease for at risk groups. Evidence that deprivation may be associated with a higher risk of retinopathy, when viewed alongside previous evidence of lower retinopathy screening uptake among deprived groups, highlights a significant potential health inequality.^56^ The national diabetic retinopathy screening programme and other stakeholders need to target and improve access to screening and support around self-management of diabetes for people living in deprived areas to avoid increasing inequalities.
