# Supplementary material for: Population trends in the 10-year incidence and prevalence of diabetic retinopathy in the UK: a cohort study in the Clinical Practice Research Datalink 2004–2014
Source: BMJ Open. 2017 Feb 28;7(2):e014444. doi: 10.1136/bmjopen-2016-014444 (PMC5337737; doi:10.1136/bmjopen-2016-014444)
Supplement: supplementary data [file bmjopen-2016-014444supp.pdf]

Supplementary Materials

Tables

Table 1. Search terms for Diabetic Retinopathy 7

Table 2. Categorization of Read codes for Diabetes Mellitus 7

Table 3. Read codes for Diabetes Mellitus 8

Table 4. Read codes for Diabetic Retinopathy diagnosis 19

Table 5.Read codes for Ethnicity (Table reproduced from http://www.clininf.eu/ethnicity.html) 22

Figures

Figure 1. Derivation of study population from the CPRD.....3

Figure 2. Results from Flowchart 1: Initial Sort and Classification.....4

Figure 3. Results from Flowchart 2: Improving classification of type 1 diabetes.....5

Figure 4. Results from Flowchart 3: Improving classification of type 2 diabetes.....6

Figure 1. Classification of Ethnicity in the CPRD
(From Mathur et al. Journal of Public Health, 2013).....25

a. Population Flow Diagram

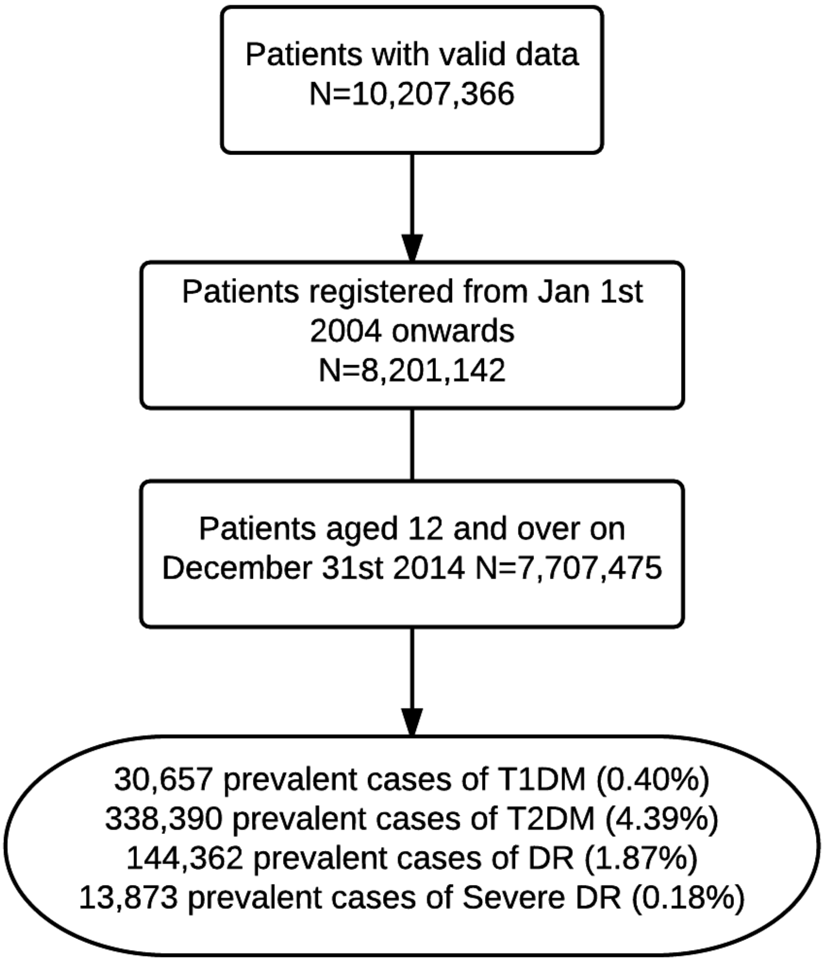

Figure 2. Derivation of study population from the CPRD

b. Results of the diabetes adjudication algorithms

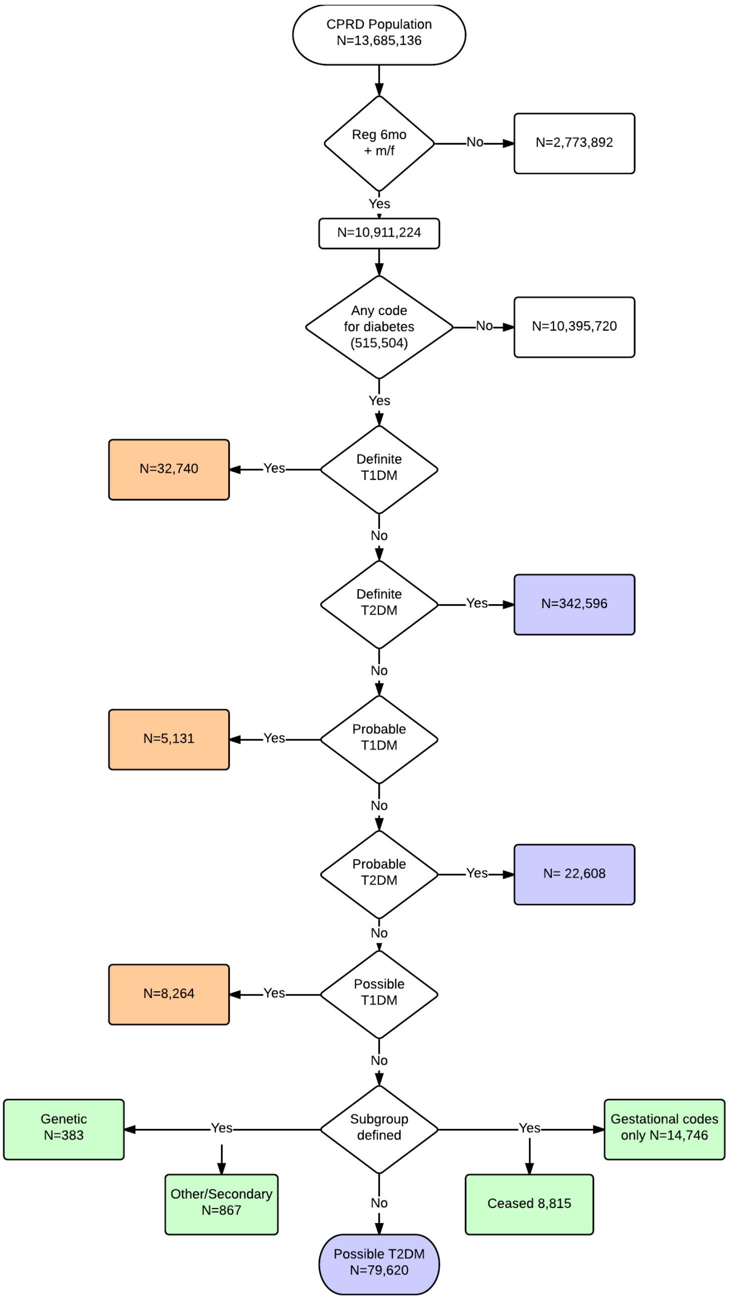

Figure 3. Results from Flowchart 1: Initial Sort and Classification

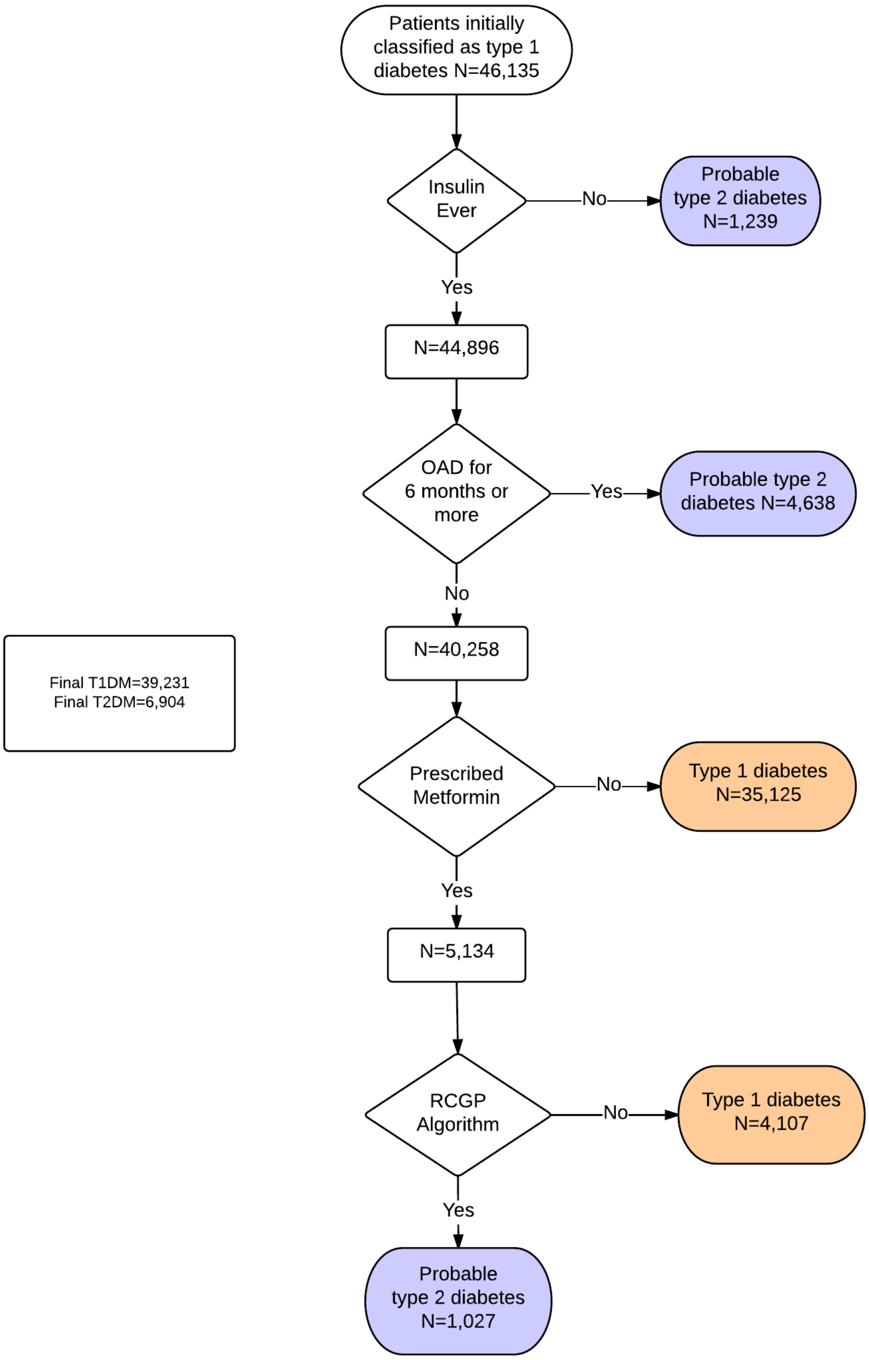

Figure 4. Results from Flowchart 2: Improving classification of type 1 diabetes

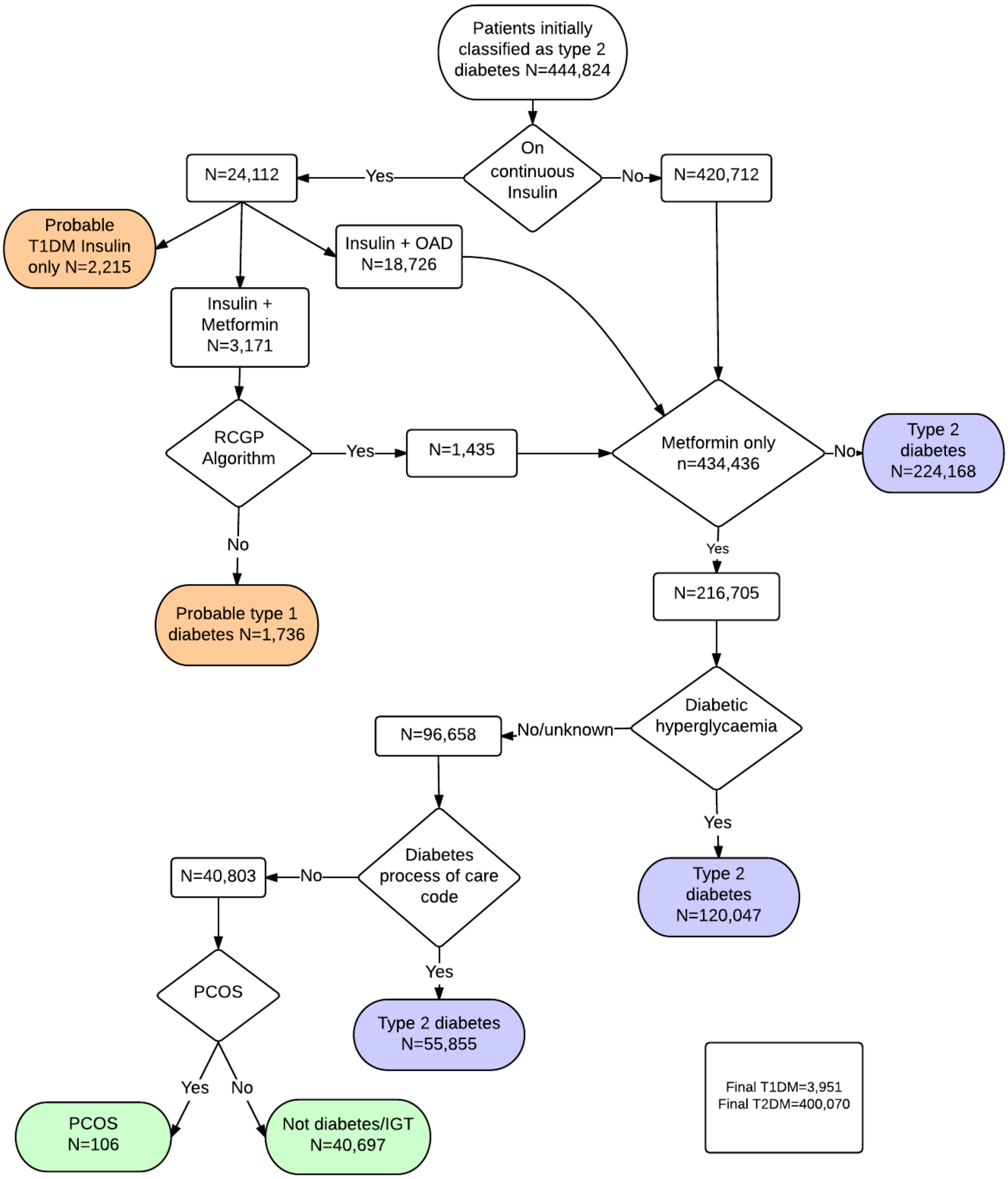

Figure 5. Results from Flowchart 3: Improving classification of type 2 diabetes

c. Codelists

Table 1. Search terms for Diabetic Retinopathy

Keywords for identifying diabetic retinopathy in the CPRD
RETINAL **and** or *SCR* or *ARTERIES* or *EXUDATE* or *MICROANEURYSMS* or *PHOTOGRAPHY* or *ABNORMALITY*
O/E **and** *RETINA* or *FUNDUS* or *PHOTOCOAGULATION* or *MACULAR* or *VITREOUS*
*LASER" **and** *RETINA*
RETINOPATHY or *FUNDOSCOPY* or *MACULOPATHY* or *RED REFLEX* or *SEEN BY OP* or *RETINAL SCR* or *RETINOSCOPY* or *SLIT LAMP* or *DIABETIC EYE* or *EYE FUNDUS* or *EXAMINATION OF RETINA* or *RETINA AND OTHER PARTS OF EYE OPERATIONS* or *VITRECTOMY*
Keywords excluded (to remove obstetric terms related to "fundus")
TERM SIZE or *WEEK SIZE* or *OBSTETRIC*

Table 2. Categorization of Read codes for Diabetes Mellitus

	Type 1 Diabetes	Type 2 Diabetes	Other
Definite	Type 1 DM: C10E<br>Not contradicted/ceased/superseded	Type 2 DM: C10F<br>Not contradicted/ceased/superseded	Gestational L180<br>Genetic C10c-C10D<br>Other/Secondary C10G-J, L-N, C11y0<br>Insulin resistance: C10K, C1098, C10F8<br>Ceased: 21263, 212H
Probable	IDDM: C108<br>Adult onset: C1073<br>Gestational: L1805<br>Not contradicted/ceased/superseded	NIDDM: C109<br>Gestational: L1806<br>Gestational: L180X<br>Not contradicted/ceased/superseded	
Possible	Diabetes mellitus, adult onset:<br>C10z1 C10y0 C110<br>Not contradicted/ceased/superseded	Diabetes mellitus, adult onset:<br>C10%, C112 (z), L180x<br>Not contradicted/ceased/superseded	

Table 3. Read codes for Diabetes Mellitus

medcode	readcode	readterm	category
28622	2126300	Diabetes resolved	Diabetes ceased
18766	212H.00	Diabetes resolved	Diabetes ceased
711	C10..00	Diabetes mellitus	Vague codes
38986	C100.00	Diabetes mellitus with no mention of complication	Vague codes
24490	C100000	Diabetes mellitus, juvenile type, no mention of complication	Possible T1 codes
1038	C100011	Insulin dependent diabetes mellitus	Possible T1 codes
14803	C100100	Diabetes mellitus, adult onset, no mention of complication	Possible T2 codes
14889	C100111	Maturity onset diabetes	Possible T2 codes
506	C100112	Non-insulin dependent diabetes mellitus	Possible T2 codes
50972	C100z00	Diabetes mellitus NOS with no mention of complication	Vague codes
1682	C101.00	Diabetes mellitus with ketoacidosis	Vague codes
53200	C101000	Diabetes mellitus, juvenile type, with ketoacidosis	Possible T1 codes
54856	C101100	Diabetes mellitus, adult onset, with ketoacidosis	Vague codes
38617	C101y00	Other specified diabetes mellitus with ketoacidosis	Vague codes
42505	C101z00	Diabetes mellitus NOS with ketoacidosis	Vague codes
21482	C102.00	Diabetes mellitus with hyperosmolar coma	Vague codes
40023	C102000	Diabetes mellitus, juvenile type, with hyperosmolar coma	Possible T1 codes
43139	C102100	Diabetes mellitus, adult onset, with hyperosmolar coma	Possible T2 codes
72345	C102z00	Diabetes mellitus NOS with hyperosmolar coma	Vague codes
15690	C103.00	Diabetes mellitus with ketoacidotic coma	Vague codes
42567	C103000	Diabetes mellitus, juvenile type, with ketoacidotic coma	Possible T1 codes
68843	C103100	Diabetes mellitus, adult onset, with ketoacidotic coma	Possible T2 codes
59288	C103y00	Other specified diabetes mellitus with coma	Vague codes
65062	C103z00	Diabetes mellitus NOS with ketoacidotic coma	Vague codes
16502	C104.00	Diabetes mellitus with renal manifestation	Vague codes
2475	C104.11	Diabetic nephropathy	Vague codes
93922	C104000	Diabetes mellitus, juvenile type, with renal manifestation	Possible T1 codes
35105	C104100	Diabetes mellitus, adult onset, with renal manifestation	Possible T2 codes
13279	C104y00	Other specified diabetes mellitus with renal complications	Vague codes

medcode	readcode	readterm	category
35107	C104z00	Diabetes mellitis with nephropathy NOS	Vague codes
33254	C105.00	Diabetes mellitus with ophthalmic manifestation	Vague codes
69748	C105000	Diabetes mellitus, juvenile type, + ophthalmic manifestation	Possible T1 codes
41389	C105100	Diabetes mellitus, adult onset, + ophthalmic manifestation	Possible T2 codes
47377	C105y00	Other specified diabetes mellitus with ophthalmic complicatn	Vague codes
34283	C105z00	Diabetes mellitus NOS with ophthalmic manifestation	Vague codes
16230	C106.00	Diabetes mellitus with neurological manifestation	Vague codes
59903	C106.11	Diabetic amyotrophy	Vague codes
7795	C106.12	Diabetes mellitus with neuropathy	Vague codes
16491	C106.13	Diabetes mellitus with polyneuropathy	Vague codes
67853	C106000	Diabetes mellitus, juvenile, + neurological manifestation	Possible T1 codes
39317	C106100	Diabetes mellitus, adult onset, + neurological manifestation	Possible T2 codes
61523	C106y00	Other specified diabetes mellitus with neurological comps	Vague codes
22573	C106z00	Diabetes mellitus NOS with neurological manifestation	Vague codes
35399	C107.00	Diabetes mellitus with peripheral circulatory disorder	Vague codes
32403	C107.11	Diabetes mellitus with gangrene	Vague codes
32556	C107.12	Diabetes with gangrene	Vague codes
70448	C107000	Diabetes mellitus, juvenile +peripheral circulatory disorder	Possible T1 codes
63357	C107100	Diabetes mellitus, adult, + peripheral circulatory disorder	Possible T2 codes
33807	C107200	Diabetes mellitus, adult with gangrene	Possible T2 codes
69124	C107300	IDDM with peripheral circulatory disorder	Probable T1 codes
56803	C107400	NIDDM with peripheral circulatory disorder	Probable T2 codes
65025	C107z00	Diabetes mellitus NOS with peripheral circulatory disorder	Vague codes
1647	C108.00	Insulin dependent diabetes mellitus	Probable T1 codes
18505	C108.11	IDDM-Insulin dependent diabetes mellitus	Probable T1 codes

medcode	readcode	readterm	category
17858	C108.12	Type 1 diabetes mellitus	Probable T1 codes
24423	C108.13	Type I diabetes mellitus	Probable T1 codes
46963	C108000	Insulin-dependent diabetes mellitus with renal complications	Probable T1 codes
61344	C108011	Type I diabetes mellitus with renal complications	Probable T1 codes
21983	C108012	Type 1 diabetes mellitus with renal complications	Probable T1 codes
49276	C108100	Insulin-dependent diabetes mellitus with ophthalmic comps	Probable T1 codes
52283	C108200	Insulin-dependent diabetes mellitus with neurological comps	Probable T1 codes
49146	C108211	Type I diabetes mellitus with neurological complications	Probable T1 codes
61829	C108212	Type 1 diabetes mellitus with neurological complications	Probable T1 codes
52104	C108300	Insulin dependent diabetes mellitus with multiple complicatn	Probable T1 codes
26855	C108400	Unstable insulin dependant diabetes mellitus	Probable T1 codes
60107	C108411	Unstable type I diabetes mellitus	Probable T1 codes
97474	C108412	Unstable type 1 diabetes mellitus	Probable T1 codes
44443	C108500	Insulin dependent diabetes mellitus with ulcer	Probable T1 codes
51957	C108511	Type I diabetes mellitus with ulcer	Probable T1 codes
68390	C108512	Type 1 diabetes mellitus with ulcer	Probable T1 codes
60499	C108600	Insulin dependent diabetes mellitus with gangrene	Probable T1 codes
6509	C108700	Insulin dependent diabetes mellitus with retinopathy	Probable T1 codes
38161	C108711	Type I diabetes mellitus with retinopathy	Probable T1 codes
41049	C108712	Type 1 diabetes mellitus with retinopathy	Probable T1 codes
6791	C108800	Insulin dependant diabetes mellitus - poor control	Probable T1 codes
46850	C108811	Type I diabetes mellitus - poor control	Probable T1 codes
45914	C108812	Type 1 diabetes mellitus - poor control	Probable T1 codes
31310	C108900	Insulin dependant diabetes maturity onset	Probable T1 codes
63017	C108911	Type I diabetes mellitus maturity onset	Probable T1 codes
97446	C108912	Type 1 diabetes mellitus maturity onset	Probable T1 codes
56448	C108A00	Insulin-dependent diabetes without complication	Probable T1 codes
95992	C108A11	Type I diabetes mellitus without complication	Probable T1 codes

medcode	readcode	readterm	category
24694	C108B00	Insulin dependent diabetes mellitus with mononeuropathy	Probable T1 codes
99231	C108B11	Type I diabetes mellitus with mononeuropathy	Probable T1 codes
41716	C108C00	Insulin dependent diabetes mellitus with polyneuropathy	Probable T1 codes
57621	C108D00	Insulin dependent diabetes mellitus with nephropathy	Probable T1 codes
66872	C108D11	Type I diabetes mellitus with nephropathy	Probable T1 codes
44440	C108E00	Insulin dependent diabetes mellitus with hypoglycaemic coma	Probable T1 codes
42729	C108E11	Type I diabetes mellitus with hypoglycaemic coma	Probable T1 codes
70766	C108E12	Type 1 diabetes mellitus with hypoglycaemic coma	Probable T1 codes
44260	C108F00	Insulin dependent diabetes mellitus with diabetic cataract	Probable T1 codes
17545	C108F11	Type I diabetes mellitus with diabetic cataract	Probable T1 codes
64446	C108G00	Insulin dependent diab mell with peripheral angiopathy	Probable T1 codes
65616	C108H00	Insulin dependent diabetes mellitus with arthropathy	Probable T1 codes
62352	C108H11	Type I diabetes mellitus with arthropathy	Probable T1 codes
39809	C108J00	Insulin dependent diab mell with neuropathic arthropathy	Probable T1 codes
60208	C108J11	Type I diabetes mellitus with neuropathic arthropathy	Probable T1 codes
18230	C108J12	Type 1 diabetes mellitus with neuropathic arthropathy	Probable T1 codes
46290	C108y00	Other specified diabetes mellitus with multiple comps	Vague codes
64449	C108z00	Unspecified diabetes mellitus with multiple complications	Vague codes
4513	C109.00	Non-insulin dependent diabetes mellitus	Probable T2 codes
5884	C109.11	NIDDM - Non-insulin dependent diabetes mellitus	Probable T2 codes
17859	C109.12	Type 2 diabetes mellitus	Probable T2 codes
18219	C109.13	Type II diabetes mellitus	Probable T2 codes
52303	C109000	Non-insulin-dependent diabetes mellitus with renal comps	Probable T2 codes
50225	C109011	Type II diabetes mellitus with renal complications	Probable T2 codes
18209	C109012	Type 2 diabetes mellitus with renal complications	Probable T2 codes
50429	C109100	Non-insulin-dependent diabetes mellitus with ophthalm comps	Probable T2 codes

medcode	readcode	readterm	category
59725	C109111	Type II diabetes mellitus with ophthalmic complications	Probable T2 codes
70316	C109112	Type 2 diabetes mellitus with ophthalmic complications	Probable T2 codes
55842	C109200	Non-insulin-dependent diabetes mellitus with neuro comps	Probable T2 codes
67905	C109211	Type II diabetes mellitus with neurological complications	Probable T2 codes
45919	C109212	Type 2 diabetes mellitus with neurological complications	Probable T2 codes
62146	C109300	Non-insulin-dependent diabetes mellitus with multiple comps	Probable T2 codes
34912	C109400	Non-insulin dependent diabetes mellitus with ulcer	Probable T2 codes
55075	C109411	Type II diabetes mellitus with ulcer	Probable T2 codes
65704	C109412	Type 2 diabetes mellitus with ulcer	Probable T2 codes
40401	C109500	Non-insulin dependent diabetes mellitus with gangrene	Probable T2 codes
62107	C109511	Type II diabetes mellitus with gangrene	Probable T2 codes
46150	C109512	Type 2 diabetes mellitus with gangrene	Probable T2 codes
17262	C109600	Non-insulin-dependent diabetes mellitus with retinopathy	Probable T2 codes
58604	C109611	Type II diabetes mellitus with retinopathy	Probable T2 codes
42762	C109612	Type 2 diabetes mellitus with retinopathy	Probable T2 codes
8403	C109700	Non-insulin dependant diabetes mellitus - poor control	Probable T2 codes
24458	C109711	Type II diabetes mellitus - poor control	Probable T2 codes
45913	C109712	Type 2 diabetes mellitus - poor control	Probable T2 codes
39406	C109800	Reaven's syndrome	Not diabetes
29979	C109900	Non-insulin-dependent diabetes mellitus without complication	Probable T2 codes
72320	C109A00	Non-insulin dependent diabetes mellitus with mononeuropathy	Probable T2 codes
50813	C109A11	Type II diabetes mellitus with mononeuropathy	Probable T2 codes
45467	C109B00	Non-insulin dependent diabetes mellitus with polyneuropathy	Probable T2 codes
47409	C109B11	Type II diabetes mellitus with polyneuropathy	Probable T2 codes
59365	C109C00	Non-insulin dependent diabetes mellitus with nephropathy	Probable T2 codes

medcode	readcode	readterm	category
64571	C109C11	Type II diabetes mellitus with nephropathy	Probable T2 codes
24836	C109C12	Type 2 diabetes mellitus with nephropathy	Probable T2 codes
43785	C109D00	Non-insulin dependent diabetes mellitus with hypoglyca coma	Probable T2 codes
56268	C109D11	Type II diabetes mellitus with hypoglycaemic coma	Probable T2 codes
61071	C109D12	Type 2 diabetes mellitus with hypoglycaemic coma	Probable T2 codes
69278	C109E00	Non-insulin depend diabetes mellitus with diabetic cataract	Probable T2 codes
48192	C109E11	Type II diabetes mellitus with diabetic cataract	Probable T2 codes
44779	C109E12	Type 2 diabetes mellitus with diabetic cataract	Probable T2 codes
54212	C109F00	Non-insulin-dependent d m with peripheral angiopath	Probable T2 codes
54899	C109F11	Type II diabetes mellitus with peripheral angiopathy	Probable T2 codes
60699	C109F12	Type 2 diabetes mellitus with peripheral angiopathy	Probable T2 codes
24693	C109G00	Non-insulin dependent diabetes mellitus with arthropathy	Probable T2 codes
18143	C109G11	Type II diabetes mellitus with arthropathy	Probable T2 codes
49869	C109G12	Type 2 diabetes mellitus with arthropathy	Probable T2 codes
40962	C109H00	Non-insulin dependent d m with neuropathic arthropathy	Probable T2 codes
47816	C109H11	Type II diabetes mellitus with neuropathic arthropathy	Probable T2 codes
66965	C109H12	Type 2 diabetes mellitus with neuropathic arthropathy	Probable T2 codes
18278	C109J00	Insulin treated Type 2 diabetes mellitus	Probable T2 codes
37648	C109J11	Insulin treated non-insulin dependent diabetes mellitus	Probable T2 codes
18264	C109J12	Insulin treated Type II diabetes mellitus	Probable T2 codes
36633	C109K00	Hyperosmolar non-ketotic state in type 2 diabetes mellitus	Probable T2 codes
52236	C10A.00	Malnutrition-related diabetes mellitus	Secondary / Other types
66675	C10A000	Malnutrition-related diabetes mellitus with coma	Secondary / Other types
33969	C10A100	Malnutrition-related diabetes mellitus with ketoacidosis	Secondary / Other types
100347	C10A500	Malnutritn-relat diabetes melitus wth periph circul complctn	Secondary / Other types
11551	C10B.00	Diabetes mellitus induced by steroids	Secondary / Other types
26108	C10B000	Steroid induced diabetes mellitus without complication	Secondary / Other types

medcode	readcode	readterm	category
43453	C10C.00	Diabetes mellitus autosomal dominant	Genetic
46624	C10C.11	Maturity onset diabetes in youth	Genetic
98392	C10C.12	Maturity onset diabetes in youth type 1	Genetic
36695	C10D.00	Diabetes mellitus autosomal dominant type 2	Genetic
59991	C10D.11	Maturity onset diabetes in youth type 2	Genetic
1549	C10E.00	Type 1 diabetes mellitus	Definite T1 codes
12455	C10E.11	Type I diabetes mellitus	Definite T1 codes
51261	C10E.12	Insulin dependent diabetes mellitus	Definite T1 codes
47582	C10E000	Type 1 diabetes mellitus with renal complications	Definite T1 codes
47649	C10E100	Type 1 diabetes mellitus with ophthalmic complications	Definite T1 codes
99311	C10E111	Type I diabetes mellitus with ophthalmic complications	Definite T1 codes
98071	C10E112	Insulin-dependent diabetes mellitus with ophthalmic comps	Definite T1 codes
42831	C10E200	Type 1 diabetes mellitus with neurological complications	Definite T1 codes
47650	C10E300	Type 1 diabetes mellitus with multiple complications	Definite T1 codes
91942	C10E311	Type I diabetes mellitus with multiple complications	Definite T1 codes
45276	C10E312	Insulin dependent diabetes mellitus with multiple complicat	Definite T1 codes
43921	C10E400	Unstable type 1 diabetes mellitus	Definite T1 codes
49949	C10E411	Unstable type I diabetes mellitus	Definite T1 codes
54600	C10E412	Unstable insulin dependent diabetes mellitus	Definite T1 codes
18683	C10E500	Type 1 diabetes mellitus with ulcer	Definite T1 codes
93878	C10E511	Type I diabetes mellitus with ulcer	Definite T1 codes
98704	C10E512	Insulin dependent diabetes mellitus with ulcer	Definite T1 codes
69993	C10E600	Type 1 diabetes mellitus with gangrene	Definite T1 codes
18387	C10E700	Type 1 diabetes mellitus with retinopathy	Definite T1 codes
95343	C10E711	Type I diabetes mellitus with retinopathy	Definite T1 codes
93875	C10E712	Insulin dependent diabetes mellitus with retinopathy	Definite T1 codes
35288	C10E800	Type 1 diabetes mellitus - poor control	Definite T1 codes
72702	C10E812	Insulin dependent diabetes mellitus - poor control	Definite T1 codes
40682	C10E900	Type 1 diabetes mellitus maturity onset	Definite T1 codes
96235	C10E911	Type I diabetes mellitus maturity onset	Definite T1 codes

medcode	readcode	readterm	category
97849	C10E912	Insulin dependent diabetes maturity onset	Definite T1 codes
69676	C10EA00	Type 1 diabetes mellitus without complication	Definite T1 codes
62613	C10EA11	Type I diabetes mellitus without complication	Definite T1 codes
99719	C10EA12	Insulin-dependent diabetes without complication	Definite T1 codes
68105	C10EB00	Type 1 diabetes mellitus with mononeuropathy	Definite T1 codes
46301	C10EC00	Type 1 diabetes mellitus with polyneuropathy	Definite T1 codes
91943	C10EC11	Type I diabetes mellitus with polyneuropathy	Definite T1 codes
101311	C10EC12	Insulin dependent diabetes mellitus with polyneuropathy	Definite T1 codes
10418	C10ED00	Type 1 diabetes mellitus with nephropathy	Definite T1 codes
39070	C10EE00	Type 1 diabetes mellitus with hypoglycaemic coma	Definite T1 codes
99716	C10EE12	Insulin dependent diabetes mellitus with hypoglycaemic coma	Definite T1 codes
49554	C10EF00	Type 1 diabetes mellitus with diabetic cataract	Definite T1 codes
100770	C10EF12	Insulin dependent diabetes mellitus with diabetic cataract	Definite T1 codes
93468	C10EG00	Type 1 diabetes mellitus with peripheral angiopathy	Definite T1 codes
18642	C10EH00	Type 1 diabetes mellitus with arthropathy	Definite T1 codes
54008	C10EJ00	Type 1 diabetes mellitus with neuropathic arthropathy	Definite T1 codes
30323	C10EK00	Type 1 diabetes mellitus with persistent proteinuria	Definite T1 codes
30294	C10EL00	Type 1 diabetes mellitus with persistent microalbuminuria	Definite T1 codes
10692	C10EM00	Type 1 diabetes mellitus with ketoacidosis	Definite T1 codes
62209	C10EM11	Type I diabetes mellitus with ketoacidosis	Definite T1 codes
40837	C10EN00	Type 1 diabetes mellitus with ketoacidotic coma	Definite T1 codes
66145	C10EN11	Type I diabetes mellitus with ketoacidotic coma	Definite T1 codes
22871	C10EP00	Type 1 diabetes mellitus with exudative maculopathy	Definite T1 codes
97894	C10EP11	Type I diabetes mellitus with exudative maculopathy	Definite T1 codes
55239	C10EQ00	Type 1 diabetes mellitus with gastroparesis	Definite T1 codes
95636	C10ER00	Latent autoimmune diabetes mellitus in adult	Secondary / Other types
758	C10F.00	Type 2 diabetes mellitus	Definite T2 codes
22884	C10F.11	Type II diabetes mellitus	Definite T2 codes
18777	C10F000	Type 2 diabetes mellitus with renal complications	Definite T2 codes

medcode	readcode	readterm	category
57278	C10F011	Type II diabetes mellitus with renal complications	Definite T2 codes
47321	C10F100	Type 2 diabetes mellitus with ophthalmic complications	Definite T2 codes
100964	C10F111	Type II diabetes mellitus with ophthalmic complications	Definite T2 codes
34268	C10F200	Type 2 diabetes mellitus with neurological complications	Definite T2 codes
98616	C10F211	Type II diabetes mellitus with neurological complications	Definite T2 codes
65267	C10F300	Type 2 diabetes mellitus with multiple complications	Definite T2 codes
43227	C10F311	Type II diabetes mellitus with multiple complications	Definite T2 codes
49074	C10F400	Type 2 diabetes mellitus with ulcer	Definite T2 codes
91646	C10F411	Type II diabetes mellitus with ulcer	Definite T2 codes
12736	C10F500	Type 2 diabetes mellitus with gangrene	Definite T2 codes
18496	C10F600	Type 2 diabetes mellitus with retinopathy	Definite T2 codes
49655	C10F611	Type II diabetes mellitus with retinopathy	Definite T2 codes
25627	C10F700	Type 2 diabetes mellitus - poor control	Definite T2 codes
47315	C10F711	Type II diabetes mellitus - poor control	Definite T2 codes
54773	C10F800	Reaven's syndrome	Not diabetes
39481	C10F811	Metabolic syndrome X	Not diabetes
47954	C10F900	Type 2 diabetes mellitus without complication	Definite T2 codes
53392	C10F911	Type II diabetes mellitus without complication	Definite T2 codes
62674	C10FA00	Type 2 diabetes mellitus with mononeuropathy	Definite T2 codes
95351	C10FA11	Type II diabetes mellitus with mononeuropathy	Definite T2 codes
18425	C10FB00	Type 2 diabetes mellitus with polyneuropathy	Definite T2 codes
50527	C10FB11	Type II diabetes mellitus with polyneuropathy	Definite T2 codes
12640	C10FC00	Type 2 diabetes mellitus with nephropathy	Definite T2 codes
46917	C10FD00	Type 2 diabetes mellitus with hypoglycaemic coma	Definite T2 codes
98723	C10FD11	Type II diabetes mellitus with hypoglycaemic coma	Definite T2 codes
44982	C10FE00	Type 2 diabetes mellitus with diabetic cataract	Definite T2 codes
93727	C10FE11	Type II diabetes mellitus with diabetic cataract	Definite T2 codes
37806	C10FF00	Type 2 diabetes mellitus with peripheral angiopathy	Definite T2 codes
59253	C10FG00	Type 2 diabetes mellitus with arthropathy	Definite T2 codes
35385	C10FH00	Type 2 diabetes mellitus with neuropathic arthropathy	Definite T2 codes
1407	C10FJ00	Insulin treated Type 2 diabetes mellitus	Definite T2 codes
64668	C10FJ11	Insulin treated Type II diabetes mellitus	Definite T2 codes

medcode	readcode	readterm	category
34450	C10FK00	Hyperosmolar non-ketotic state in type 2 diabetes mellitus	Definite T2 codes
26054	C10FL00	Type 2 diabetes mellitus with persistent proteinuria	Definite T2 codes
60796	C10FL11	Type II diabetes mellitus with persistent proteinuria	Definite T2 codes
18390	C10FM00	Type 2 diabetes mellitus with persistent microalbuminuria	Definite T2 codes
85991	C10FM11	Type II diabetes mellitus with persistent microalbuminuria	Definite T2 codes
32627	C10FN00	Type 2 diabetes mellitus with ketoacidosis	Definite T2 codes
51756	C10FP00	Type 2 diabetes mellitus with ketoacidotic coma	Definite T2 codes
25591	C10FQ00	Type 2 diabetes mellitus with exudative maculopathy	Definite T2 codes
63690	C10FR00	Type 2 diabetes mellitus with gastroparesis	Definite T2 codes
95539	C10FS00	Maternally inherited diabetes mellitus	Genetic
51697	C10G.00	Secondary pancreatic diabetes mellitus	Secondary / Other types
96506	C10G000	Secondary pancreatic diabetes mellitus without complication	Secondary / Other types
61122	C10H.00	Diabetes mellitus induced by non-steroid drugs	Secondary / Other types
67212	C10H000	DM induced by non-steroid drugs without complication	Secondary / Other types
68517	C10J.00	Insulin autoimmune syndrome	Secondary / Other types
37957	C10K.00	Type A insulin resistance	Not diabetes
56885	C10K000	Type A insulin resistance without complication	Not diabetes
43857	C10M.00	Lipoatrophic diabetes mellitus	Secondary / Other types
22487	C10N.00	Secondary diabetes mellitus	Secondary / Other types
94383	C10N000	Secondary diabetes mellitus without complication	Secondary / Other types
93380	C10N100	Cystic fibrosis related diabetes mellitus	Secondary / Other types
33343	C10y.00	Diabetes mellitus with other specified manifestation	Vague codes
63371	C10y100	Diabetes mellitus, adult, + other specified manifestation	Probable T2 codes
10098	C10yy00	Other specified diabetes mellitus with other spec comps	Vague codes
70821	C10yz00	Diabetes mellitus NOS with other specified manifestation	Vague codes
45491	C10z.00	Diabetes mellitus with unspecified complication	Vague codes
68792	C10z000	Diabetes mellitus, juvenile type, + unspecified complication	Possible T1 codes

medcode	readcode	readterm	category
63762	C10z100	Diabetes mellitus, adult onset, + unspecified complication	Probable T2 codes
64283	C10zy00	Other specified diabetes mellitus with unspecified comps	Vague codes
64357	C10zz00	Diabetes mellitus NOS with unspecified complication	Vague codes
2472	C110.00	Hypoglycaemic coma	Possible T1 codes
53630	C110.11	Insulin coma	Possible T1 codes
61520	C110000	Iatrogenic hyperinsulinism	Secondary / Other types
72882	C110100	Self-induced hyperinsulinism	Probable T1 codes
51371	C110z00	Hypoglycaemic coma NOS	Possible T1 codes
1410	C112.00	Hypoglycaemia unspecified	Possible T2 codes
4563	C112000	Reactive hypoglycaemia NOS	Possible T2 codes
24405	C112100	Spontaneous hypoglycaemia NOS	Possible T2 codes
20368	C112z00	Hypoglycaemia unspecified NOS	Possible T2 codes
11359	L180.00	Diabetes mellitus during pregnancy/childbirth/puerperium	Probable Gestational diabetes
67635	L180000	Diabetes mellitus - unspec whether in pregnancy/puerperium	Probable Gestational diabetes
34639	L180100	Diabetes mellitus during pregnancy - baby delivered	Probable Gestational diabetes
49559	L180300	Diabetes mellitus during pregnancy - baby not yet delivered	Probable Gestational diabetes
96823	L180400	Diabetes mellitus in pueperium - baby previously delivered	Probable T1 codes
50960	L180500	Pre-existing diabetes mellitus, insulin-dependent	Probable T1 codes
50609	L180600	Pre-existing diabetes mellitus, non-insulin-dependent	Probable T2 codes
10278	L180800	Diabetes mellitus arising in pregnancy	Probable Gestational diabetes
8446	L180811	Gestational diabetes mellitus	Probable Gestational diabetes
2664	L180900	Gestational diabetes mellitus	Probable Gestational diabetes
55431	L180X00	Pre-existing diabetes mellitus, unspecified	Vague codes
64384	L180z00	Diabetes mellitus in pregnancy/childbirth/puerperium NOS	Probable Gestational diabetes

Table 4. Read codes for Diabetic Retinopathy diagnosis and screening

medcode	readcode	readterm	DR category
52041	2BBl.00	O/E - left eye stable treated prolif diabetic retinopathy	DR
52630	2BBo.00	O/E - sight threatening diabetic retinopathy	DR
19533	2BBY.00	O/E - referable retinopathy	DR
3837	F420400	Diabetic maculopathy	DR
47328	2BBk.00	O/E - right eye stable treated prolif diabetic retinopathy	DR
101881	2BBr.00	Impaired vision due to diabetic retinopathy	DR
3914	2BB9.00	O/E - retinal pigmentation	DR
9339	F421.00	Other background retinopathy	DR
10882	F421400	Exudative retinopathy	DR
48751	2BB3.00	O/E - retinal A-V nipping	DR
42762	C109612	Type 2 diabetes mellitus with retinopathy	DR
35659	2BB7.00	O/E - retinal vascular prolif.	DR
38161	C108711	Type I diabetes mellitus with retinopathy	DR
72424	7270B00	Vitrectomy using anterior approach	DR
9835	2BBL.00	O/E - diabetic maculopathy present both eyes	DR
39457	F421C00	Other intraretinal microvascular abnormality	DR
55026	7270B11	Anterior vitrectomy	DR
11053	F421800	Retinal microaneurysms NOS	DR
18387	C10E700	Type 1 diabetes mellitus with retinopathy	DR
4514	7270011	Anterior vitrectomy	DR
13102	2BBW.00	O/E - right eye diabetic maculopathy	DR
13108	2BBX.00	O/E - left eye diabetic maculopathy	DR
36119	F421111	Arterosclerotic retinopathy	DR
93875	C10E712	Insulin dependent diabetes mellitus with retinopathy	DR
8595	F42y600	Retinal exudate or deposit	DR
102242	2BBs.00	Retinal arteries silverwire	DR
17916	F422011	Retinopathy of prematurity	DR
22871	C10EP00	Type 1 diabetes mellitus with exudative maculopathy	DR
1411	3128100	Fundoscopy abnormal	DR
11626	F420z00	Diabetic retinopathy NOS	DR
34455	F421112	Atheroscleritic retinopathy	DR
66964	F426500	Pseudoretinitis pigmentosa	DR
2254	F424100	Central serous retinopathy	DR
36867	2BBa.00	O/E- non-referable retinopathy	DR
11129	2BBQ.00	O/E - left eye background diabetic retinopathy	DR
88368	7270411	Vitrectomy using pars plana approach	DR
6509	C108700	Insulin dependent diabetes mellitus with retinopathy	DR
45876	F421200	Renal retinopathy	DR
8742	2BB5.00	O/E - retinal haemorrhages	DR
17262	C109600	Non-insulin-dependent diabetes mellitus with retinopathy	DR
13107	2BBn.00	O/E - left eye clinically significant macular oedema	DR
104263	F425900	Maculopathy	DR

58604	C109611	Type II diabetes mellitus with retinopathy	DR
41049	C108712	Type 1 diabetes mellitus with retinopathy	DR
1323	F420.00	Diabetic retinopathy	DR
40982	F421z00	Other background retinopathy NOS	DR
50656	2BBc.00	O/E - No retinal laser photocoagulation scars	DR
36855	2BBG.00	Retinal abnormality - non-diabetes	DR
3822	2BB8.00	O/E - vitreous haemorrhages	DR
49655	C10F611	Type II diabetes mellitus with retinopathy	DR
11433	2BBP.00	O/E - right eye background diabetic retinopathy	DR
17293	727..00	Retina and other parts of eye operations	DR
69662	F421G00	Venostasis retinopathy	DR
7069	F420000	Background diabetic retinopathy	DR
1438	F421000	Unspecified background retinopathy	DR
97894	C10EP11	Type I diabetes mellitus with exudative maculopathy	DR
13106	2BB6.00	O/E - retinal exudates	DR
22967	2BBF.00	Retinal abnormality - diabetes related	DR
25888	2BBm.00	O/E - right eye clinically significant macular oedema	DR
25591	C10FQ00	Type 2 diabetes mellitus with exudative maculopathy	DR
18496	C10F600	Type 2 diabetes mellitus with retinopathy	DR
31829	F433100	Solar retinopathy	DR
41229	F421100	Atherosclerotic retinopathy	DR
19532	2BB4.00	O/E - retinal microaneurysms	DR
95343	C10E711	Type I diabetes mellitus with retinopathy	DR
11858	7270400	Pars plana vitrectomy	DR
6702	F421300	Hypertensive retinopathy	DR
45145	2BB2.00	O/E - retinal vessel narrowing	DR
2986	F420200	Preproliferative diabetic retinopathy	DR
13103	2BBS.00	O/E - left eye preproliferative diabetic retinopathy	DR
13099	2BBR.00	O/E - right eye preproliferative diabetic retinopathy	DR
10755	F420600	Non proliferative diabetic retinopathy	DR
65463	F420800	High risk non proliferative diabetic retinopathy	DR
27022	5B42.00	Laser therapy - retinal lesion	Severe DR
86068	7272800	Panretinal laser photocoagulation to lesion of retina	Severe DR
13097	2BBT.00	O/E - right eye proliferative diabetic retinopathy	Severe DR
100979	7272900	Focal laser photocoagulation of retina	Severe DR
11874	F422100	Proliferative retinopathy due to sickle cell disease	Severe DR
96926	FyuF700	[X]Other proliferative retinopathy	Severe DR
6836	7271100	Laser photocoagulation of retina for detachment	Severe DR
11912	5B4..11	Retinal laser therapy	Severe DR
30477	F420700	High risk proliferative diabetic retinopathy	Severe DR
9318	7272300	Laser destruction of lesion of retina	Severe DR
36035	F422y00	Other specified other proliferative retinopathy	Severe DR
18775	2BBO.00	O/E - Laser photocoagulation scars	Severe DR
10099	F420300	Advanced diabetic maculopathy	Severe DR
46068	7272500	Panretinal laser photocoagulation to lesion of retina	Severe DR

		NEC	
13101	2BBV.00	O/E - left eye proliferative diabetic retinopathy	Severe DR
38096	F422z00	Proliferative retinopathy NOS	Severe DR
3286	F420100	Proliferative diabetic retinopathy	Severe DR
7890	F422.00	Other proliferative retinopathy	Severe DR
881	3128	Fundoscopy	Screening
19535	2BBA.00	Examination of retina	Screening
10701	8HBD.00	Retinopathy follow up	Screening
25116	2BBZ.00	O/E - retinal inspection NOS	Screening
92317	2BBf.00	O/E - left retina partially assessable	Screening
18311	68A7.00	Diabetic retinopathy screening	Screening
106269	9m0..00	Diabetic retinopathy screening administrative status	Screening
33681	2BB..00	O/E - retinal inspection	Screening
17871	312E.00	Direct fundoscopy following mydriatic	Screening
17198	2BB..11	O/E - retina	Screening
19531	3128.11	Retinoscopy	Screening
13196	66AD.00	Fundoscopy - diabetic check	Screening
36619	312F.00	Camera fundoscopy	Screening
19534	3128300	Camera fundoscopy	Screening
11891	68A8.00	Digital retinal screening	Screening
9974	9N1v.00	Seen in diabetic eye clinic	Screening
70163	2BBe.00	O/E - right retina partially assessable	Screening
8140	9N2V.00	Seen by optometrist	Screening
66273	2BBg.00	O/E - right retina fully assessable	Screening
61021	68AB.00	Diabetic digital retinopathy screening offered	Screening
13105	58C1.00	Retinal photography	Screening
13098	3128Z00	Fundoscopy NOS	Screening
30111	3129	Eye fundus photography	Screening
20991	312A.00	Slit lamp examination	Screening
12528	9NNC.00	Under care of retinal screener	Screening
18662	8HBH.00	Diabetic retinopathy 6 month review	Screening
12636	9N2f.00	Seen by retinal screener	Screening
9934	9N2e.00	Seen by ophthalmologist	Screening
11018	8HBG.00	Diabetic retinopathy 12 month review	Screening
22966	3128400	Indirect fundoscopy following mydriatic	Screening
6108	9N2U.00	Seen by optician	Screening
64070	312G.00	Indirect fundoscopy following mydriatic	Screening
95916	2BBh.00	O/E - left retina fully assessable	Screening

Table 5.Read codes for Ethnicity (Table reproduced from http://www.clininf.eu/ethnicity.html)

Grouping of the 9S and 9i ethnic codes to the '16+1' format and the five category classifications			
**Five category**	**16 category framework**	**9i… Ethnic category hierarchy**	**9S.. Ethnic group hierarchy**
1. White	1. British or Mixed British	**9i0** British or mixed British	**9S1** White, **9S10** White British, **9S14** Other white British ethnic grp
	2. Irish	**9i1** Irish	**9S11** White Irish, **9SA9** Irish NMO, **9SI** Irish traveller
	3. Other White	**9i2** Other White	**9S12** Other white ethnic group
		**9i20** English	
		**9i21** Scottish	**9S13** White Scottish
		**9i22** Welsh, **9i26** Cypriot part unsp, **9i27** Greek, **9i28** Greek Cypriot, **9i29** Turkish, **9i2A** Turkish Cypriot, **9i2B** Italian, **9i2C** Irish Traveller, **9i2D** Traveller, **9i2E** Gypsy/Romany, **9i2F** Polish, **9i2H**Commonwealth of (Russian), **9i2J** Kosovan, **9i2K** Albanian Serbian, **9i2P** Oth repub Yugoslav, **9i2R** Oth White/unsp/Mix Eur,**9i2S** Oth mixed White, **9i2T** Other White or White unspecified.	
2. Mixed	4. White + Black Caribbean	**9i3** White & Black Caribbean	**9SB5** Black Caribbean and White
	5. White + Black African	**9i4** White and Black African	**9SB6** Black African and White
	6. White + Asian	**9i5** White & Asian	**9SB2** Other ethnic, Asian/White orig
	7. Other mixed	**9i6** Other Mixed	**9SB** Other ethnic, mixed origin , **9SB3** Other ethnic, mixed white orig, **9SB4** Other ethnic, other mixed

			orig,**9S52** Other Black - Black/Asian orig.
		**9i60** Black & Asian, **9i61** Black & Chinese	
		**9i62** Black and White	**9SB1** Other ethnic, Black/White orig, **9S51** Other Black – Black/White orig
		**9i63** Chinese & White, **9i64** Asian & Chinese	
3. Asian or Asian British	8. Indian or British Indian	**9i7** Indian/British Indians	**9S6** Indian
	9. Pakistani or British Pakistani	**9i8** Pakistani/Brit Pakists	**9S7** Pakistani
	10. Bangladeshi or British Bangladeshi	**9i9** Bangladeshi/Brit Bangl	**9S8** Bangladeshi
	11. Other Asian	**9iA** Other Asian	**9SH** Other Asian ethnic group, **9SA8** Other Asian NMO, **9SA7** Indian sub-continent NMO
		**9iA3** East African Asian	**9SA6** E Afric Asian/Indo-Carib NMO
		**9iA4** Sri Lankan, **9iA5** Tamil, **9iA6** Sinhalese, **9iA7** Carib Asian,**9iA8** Briti Asian, **9iA9** Mixed Asian	
4. Other Black	12. Caribbean	**9iB** Caribbean	**9S2** Black Caribbean
	13. African	**9iC** African	**9S3** Black African, **9S44**Black - other African country,**9SA5** Other African countries NMO
	14 Other Black	**9iD** Other Black	**9S4** Black, other, non-mixed origin, **9S42** Black Caribbean/W.I./ Guyana, **9S43** Black N African/Arab/Ira

			nian, **9S45** Black E Afric Asia/Indo-Caribb, **9SG** Other black ethnic group, **9S47** Black - other Asian, **9S48** Black Black - other, **9S5** Black - other, mixed, **9SA3** Caribbean I./W.I./Guyana NMO
		**9iD0** Somali, **9iD1** Nigerian	
		**9iD2** Black British	**9S41** Black British
5. Other ethnic groups	15. Chinese	**9iE** Chinese	**9S9** Chinese
	16. Other	**9iF** Other	**9SJ** Other ethnic group, **9SA**Other ethnic non-mixed NMO, **9SA2** Brit. ethnic minor. unsp NMO, **9SAA**Greek/Greek Cypriot NMO,**9SAB** Turkish/Turkish Cypriot NMO **9SAC** Other European NMO,**9SAD** Other ethnic NEC NMO
		**9iF0** Vietnamese	**9SC** Vietnamese
		**9iF1** Japanese, **9iF2** Filipino, **9iF3** Malaysian, **9iF9** Arab	
		**9iFA** North African	**9SA4** N African Arab/Iranian NMO
		**9iFB** ME ex Isr/Iran/Arab, **9iFD** Iranian, **9iFE** Kurdish, **9iFG** Latin American, **9iFH** South/Central American, **9iFJ** Multi-ethnic islands: Mauritian or Seychellois or Maldivian or St Helena, **9iFK** Any other - ethn categ	

6. Not stated	17. (16+1)<br><br>Not stated	**9iG** Ethn cat not stated	**9S** Ethnic groups census,**9SD** Ethnic group - patient refused, **9SE** Ethnic group not recorded, **9SZ** Ethnic groups census NOS

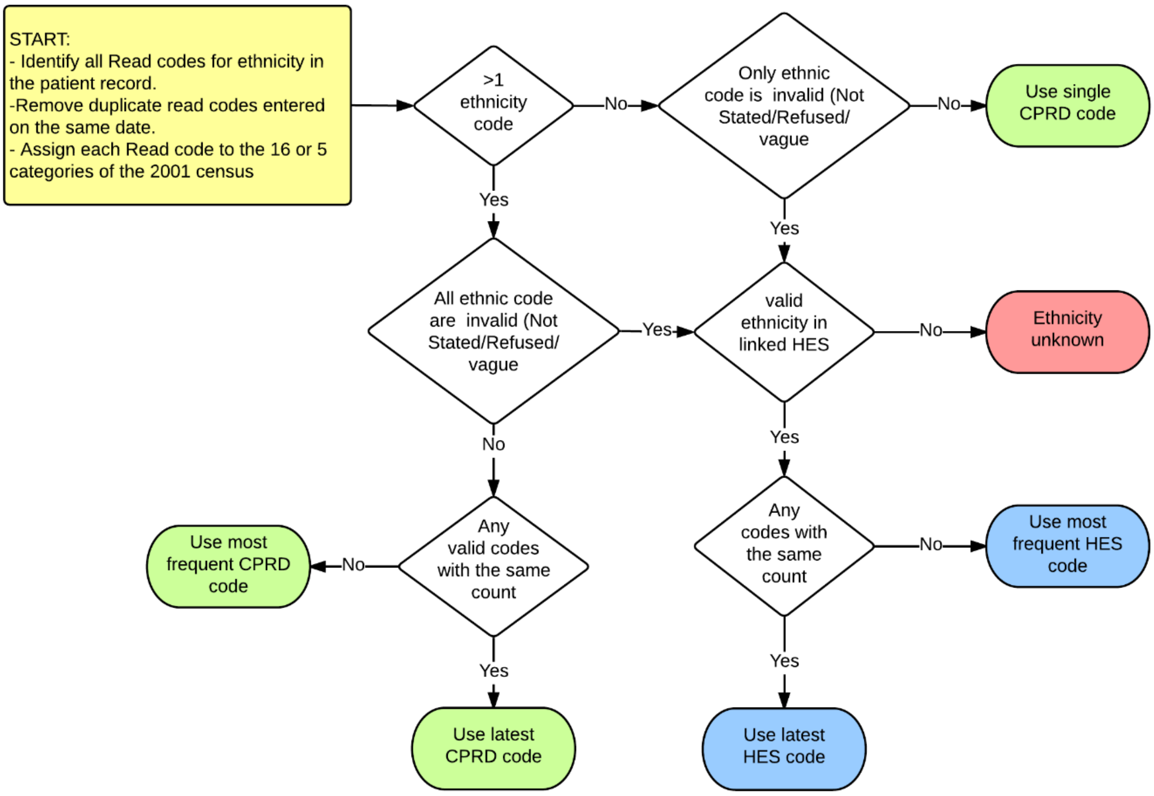

Figure 6. Classification of Ethnicity in the CPRD (From Mathur et al. Journal of Public Health, 2013)
